# New records of wild bees from a spring expedition in southern Türkiye, with six new species for the country (Hymenoptera, Apoidea, Anthophila)

**DOI:** 10.3897/BDJ.14.e191847

**Published:** 2026-06-29

**Authors:** Rémi Santerre, Denis Michez, Ahmet Murat Aytekin, Seçil Aytekin, Jordan Benrezkallah, Thomas Brau, Frédéric Carion, Fatih Dikmen, Achik Dorchin, Vahap Eldem, Simone Flaminio, Sebastian Hopfenmüller, Ece Kamalak, Anatole Maugendre, Çiğdem Özenirler, Pierre Rasmont, Blandine Rigaut, Michaël Terzo, Clément Tourbez, Guillaume Ghisbain

**Affiliations:** 1 Laboratory of Zoology, Research Institute for Biosciences, University of Mons, Mons, Belgium Laboratory of Zoology, Research Institute for Biosciences, University of Mons Mons Belgium; 2 Department of Biology, Faculty of Science, Hacettepe University, Beytepe (Ankara), Türkiye Department of Biology, Faculty of Science, Hacettepe University Beytepe (Ankara) Türkiye; 3 Turkish Doping Control Center, Hacettepe University, Sıhhiye (Ankara), Türkiye Turkish Doping Control Center, Hacettepe University Sıhhiye (Ankara) Türkiye; 4 Department of Biology, Faculty of Science, Istanbul University, Istanbul, Türkiye Department of Biology, Faculty of Science, Istanbul University Istanbul Türkiye; 5 Stiftung Kulturlandschaft Günztal, Ottobeuren, Germany Stiftung Kulturlandschaft Günztal Ottobeuren Germany

**Keywords:** Anthophila, biodiversity, distribution, occurrence, Türkiye, wild bees

## Abstract

**Background:**

Bees (Anthophila) are key pollinators, yet major gaps persist in the knowledge of their diversity and distribution, particularly across Mediterranean biodiversity hotspots. Türkiye, located at the intersection of Europe and Asia, hosts amongst the richest bee faunas in the Western Palaearctic, with more than 1,800 recorded species. Despite previous faunistic work, the national fauna remains incompletely documented, as shown by numerous recent species descriptions and new country records. Additional sampling is, therefore, required to improve taxonomic and distributional knowledge and to support biodiversity research and conservation in this highly diversified region.

**New information:**

The dataset comprises 2,070 wild bee specimens collected in southern Türkiye, with 246 identified species. Amongst these, six species are recorded for the first time from Türkiye and several additional noteworthy species of taxonomic, biogeographical or conservation interest are highlighted and illustrated. This dataset provides new, well-documented occurrence records contributing to an improved understanding of bee diversity in the region.

## Introduction

In recent decades, the growing awareness of threats to biodiversity in the Anthropocene has brought certain groups of living organisms, such as bees (Anthophila), to the forefront (Brown and Paxton 2009, Potts et al. 2010, Potts et al. 2016, Kammerer et al. 2021, Gekière et al. 2025). Through their major contribution to the pollination of wildflowers and crops, these insects represent a cornerstone of ecosystem sustainability and global food security (Klein et al. 2006, Devkota et al. 2024). However, even in the comparatively well-studied Western Palaearctic Region, several key elements are still missing about their distribution and taxonomy, thereby hindering conservation efforts (Marshall et al. 2024, Ghisbain et al. 2025, Michez et al. 2026). These knowledge gaps are even more pronounced in regions with highly diversified faunas, such as the Mediterranean Basin (Marshall et al. 2024, Michez et al. 2026). Amongst these biodiversity hotspots, Türkiye benefits from a wide diversity of habitats and climates and occupies a special geographical position influenced by both Asian and European faunas and floras, with a high level of bee endemism presumably linked to the remarkably high level of plant endemism in the region (Türe and Böcük 2010, Şekercioğlu et al. 2011, Rasmont et al. 2021).

With over 1,800 bee species recorded, Türkiye is amongst the most diverse regions in the Western Palaearctic and represents a centre of diversity and a presumed region of origin for several bee groups (Wood 2020, Pisanty and Wood 2024, Straka et al. 2024, Ascher and Pickering 2025). Although the region has been partially covered by some earlier broad-scale revisions, the first detailed faunistic surveys were provided by Klaus Warncke who intensively and repeatedly collected across the country (e.g. Warncke (1965), Warncke (1974), Warncke (1975a), Warncke (1975b), Warncke (1984), Warncke (1988), Warncke (1992)). More recently, a number of taxon-focused surveys at regional scale have kept updating our knowlegde of the composition and distribution of the Turkish fauna (e.g. Kuhlmann and Özbek (2007), Dikmen et al. (2011), Güler et al. (2014), Hazir et al. (2014), Özbek (2014), Özbek et al. (2015), Özbek and Terzo (2016), Özbek and Dathe (2020)). However, our knowledge remains broadly incomplete (even in Eastern Thrace), as evidenced by the dozens of species that have been described or newly reported from the country over the last years (e.g. Scheuchl and Hazir (2012), Müller (2015), Pauly et al. (2015), Bogusch (2018), Dorchin and Praz (2018), Smit (2018), Dorchin (2019), Schwarz et al. (2019), Wood (2020), Wood (2021), Wood (2023), Aubert et al. (2024b), Pisanty and Wood (2024), Wood (2024a), Wood (2024b), Wood (2025)). Therefore, renewed sampling efforts are required to further support ongoing taxonomic research and to achieve a more comprehensive understanding of Türkiye’s remarkable bee diversity. This article presents the data associated with wild bee specimens collected during a 10-days sampling expedition across southern Türkiye in Spring 2025.

## Materials and methods

### Study area

**Figure 1.**
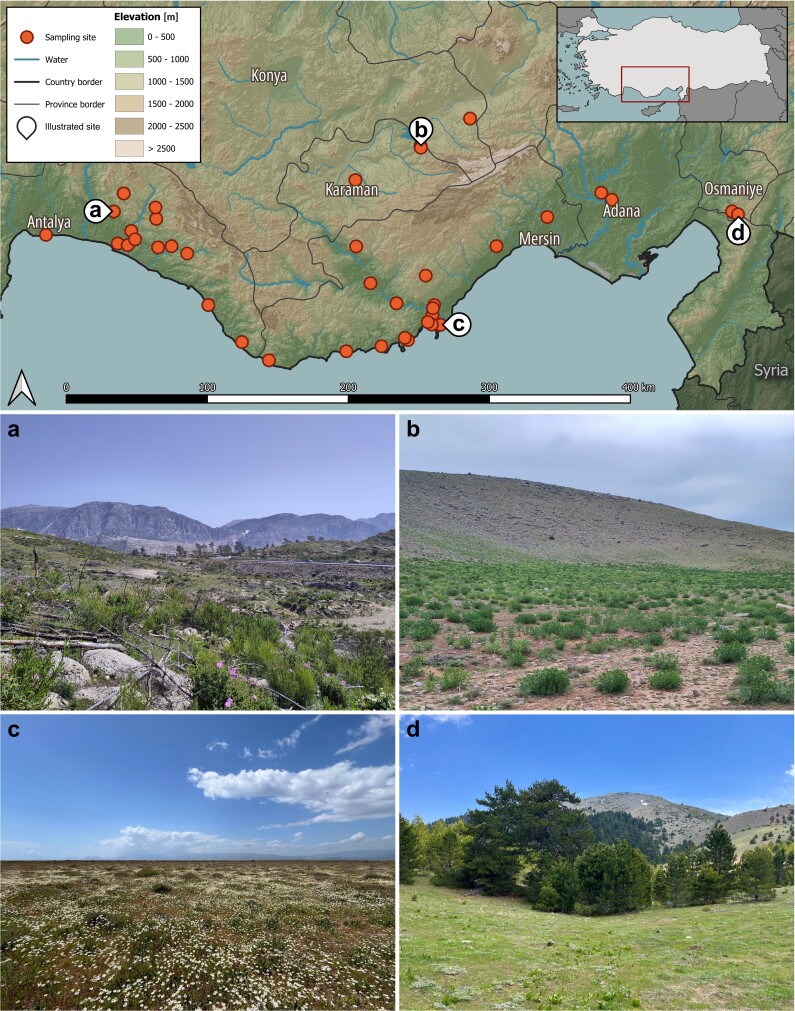
Map of the sites where sampling was undertaken **a** Mediterranean shrubland near Beydiğin (Antalya Province); **b** Steppe near Böğecik (Karaman Province); **c** Halophilic grassland near Sökün (Mersin Province); **d** Subalpine grassland in Nur Mountains (Osmaniye Province). Photo credit D. Michez (a, b) & G. Ghisbain (c, d). Map compiled by J. Benrezkallah using QGIS v.3.40. Boundaries: JRC (Urbano 2018). Topography: SRTM 90 m (Reuter et al. 2007). Hydrography: Copernicus (European Union's Copernicus Land Monitoring Service information 2020); HDX (Humanitarian Data Exchange 2025). Projection: TUREF / TM33 (EPSG:5255).

The sampling was carried out across 42 sites distributed across southern Türkiye (Fig. 1): 38 were located in the Mediterranean Region (provinces: Adana, Antalya, Mersin, Osmaniye) and four in the south of the Central Anatolia Region (provinces: Karaman, Konya). A broad variety of habitats and vegetation types were therefore covered, such as Mediterranean shrublands (Fig. 1a), steppe (Fig. 1b), marshes and halophilic grasslands (Fig. 1c) and subalpine and alpine grasslands (Fig. 1d).

The selection of sampling sites was done using searches in Google Maps for potential high-quality habitats (mainly sites surrounded by ample natural habitats), followed by on-site surveys to confirm the presence of a sufficient floral diversity for attracting a high diversity of bees.

### Sampling process

Wild bees were collected using hand nets, trying to prioritise the species diversity by sampling on a maximum diversity of flowering plants (no standardised sampling protocol). Samplings were generally carried on from 9 a.m. to 5 p.m. Specimens were killed with cyanide or ethyl acetate and pinned in collection the same day. Each specimen was labelled with the main collection information (i.e. Locality, altitude, GPS coordinates, Date, Collector) and with a unique identification code added to the same label.

### Specimen identification and quality control

Species-level identification was performed in the lab by Thomas Brau (*Halictus*, *Seladonia*, *Thrincohalictus*), Frédéric Carion (*Nomada*), Achik Dorchin (*Eucera*), Simone Flaminio (*Ceylalictus*, *Lasioglossum*, *Rophites*), Guillaume Ghisbain (*Bombus*, *Dasypoda*), Sebastian Hopfenmüller (*Panurginus*), Michael Kuhlmann (*Colletes*), Romain Le Divelec (*Hylaeus*, *Sphecodes*), Anatole Maugendre (*Coelioxys*), Denis Michez (*Dasypoda*), Andreas Müller (*Chelostoma*, *Hoplitis*, *Osmia*), Pierre Rasmont (*Amegilla*, *Anthophora*, *Eupavlovskia*, *Habropoda*), Blandine Rigaut (*Dufourea*), Rémi Santerre (*Ammobatoides*, *Anthophora*, *Ceratina*, *Melitturga*, *Nomiapis*), Jakub Straka (*Nomada*, *Sphecodes*), Michaël Terzo (*Ceratina*, *Xylocopa*), Clément Tourbez (*Anthidiellum*, *Anthidium*, *Chelostoma*, *Heriades*, *Hoplitis*, *Megachile*, *Osmia*, *Protosmia*, *Pseudoanthidium*, *Rhodanthidium*, *Stelis*, *Stenoheriades*) and Thomas J. Wood (*Andrena*, *Camptopoeum*, *Panurgus*). Reference material from the collection of the Laboratory of Zoology (University of Mons, Belgium) and from each expert's personal research collection was used to achieve the identifications. Where conflicting identifications were provided by different experts, these were specified in the ‘identificationRemarks’ column of the dataset (Suppl. material 1).

The geographical consistency of the identifications was first verified for each species using centralised resources (e.g. Rasmont (2014), Ascher and Pickering (2025), Müller (2025)), then additional targeted documentation was sought and a double-check of the identifications was carried out by Rémi Santerre to confirm the validity of the taxa found outside of their known range.

The species of particular interest that are highlighted below were illustrated through photographies taken with Olympus OM-D E-M1 Mark II, equipped with a M.Zuiko Digital ED 60 mm f/2.8 Macro lens and stacked with Helicon Focus 8.2 (Helicon Soft Ltd, Kharkiv, Ukraine) (pictures with grey background). Additional close-up images (with white background) were captured through automatic stacking with Keyence VHX-970F digital microscope, equipped with a VHX-7020 camera and a VH-Z20R/Z20T zoom lens.

## Data resources

The complete dataset is included as supplementary material (Suppl. material 1) and an online version (subject to future updates) is publicly available online at https://doi.org/10.15468/jre3sd (Santerre and Benrezkallah 2026).

### Summary of the taxa collected

The dataset covers 246 confidently identified species of wild bees, belonging to the six bee families present in the Palaearctic Region (Michener 2007): Andrenidae, Apidae, Colletidae, Halictidae, Megachilidae and Melittidae (Hymenoptera, Apoidea, Anthophila). It includes another seven taxa for which the uncertain species identity is marked by the notation 'cf.' (uncertain identification), 'aff.' (belongs to a taxon closely related, but distinct from the mentioned species) or 'gr.' (belongs to one of the species from the species-group mentioned). In addition, 112 specimens in this dataset remain unidentified, representing between 27 and 42 recognisable taxonomic units (RTUs), some of which may correspond to species already identified. These specimens are distributed amongst the following genera: *Amegilla* (7 specimens, 2 RTUs), *Andrena* (26 specimens, 10–23 RTUs), *Anthophora* (5 specimens, 2–3 RTUs), *Chelostoma* (11 specimens, 1–2 RTUs), *Eucera* (21 specimens, 3 RTUs), *Halictus* (29 specimens, 6 RTUs), *Lasioglossum* (12 specimens, 2 RTUs) and *Seladonia* (1 specimen, 1 RTU) (Table 1).

## Checklists

### Species new for the fauna of Türkiye

#### Andrena igraeca

Pisanty & Wood, 2022

6E07EBB9-5068-5A86-B61E-53805A4BEE16

##### Materials

- **Type status:** Other material. **Occurrence:** recordedBy: G. Ghisbain, D. Michez & R. Santerre; sex: 5 females; occurrenceID: E5B5D02E-DC36-5CB3-8251-7BA89D4DC432; **Location:** country: Türkiye; stateProvince: Osmaniye; locality: Dereobası; verbatimElevation: 1412 m; decimalLatitude: 36.97654; decimalLongitude: 36.32773; **Event:** eventDate: 28/04/2025
- **Type status:** Other material. **Occurrence:** recordedBy: G. Ghisbain, D. Michez & R. Santerre; sex: 3 females, 1 male; occurrenceID: 22C706E5-CC4A-5307-BCAC-A0F7B3F505D4; **Location:** country: Türkiye; stateProvince: Osmaniye; locality: Dereobası; verbatimElevation: 1926 m; decimalLatitude: 36.96265; decimalLongitude: 36.36852; **Event:** eventDate: 28/04/2025
- **Type status:** Other material. **Occurrence:** recordedBy: G. Ghisbain, D. Michez & R. Santerre; sex: 3 females, 1 male; occurrenceID: BADB6FBD-1885-5C90-AFC0-1ADB7D98A3E7; **Location:** country: Türkiye; stateProvince: Osmaniye; locality: Dereobası; verbatimElevation: 1346 m; decimalLatitude: 36.98222; decimalLongitude: 36.32254; **Event:** eventDate: 28/04/2025

##### Distribution

Levant (Pisanty et al. 2022).

##### Notes

**Figure 2.**
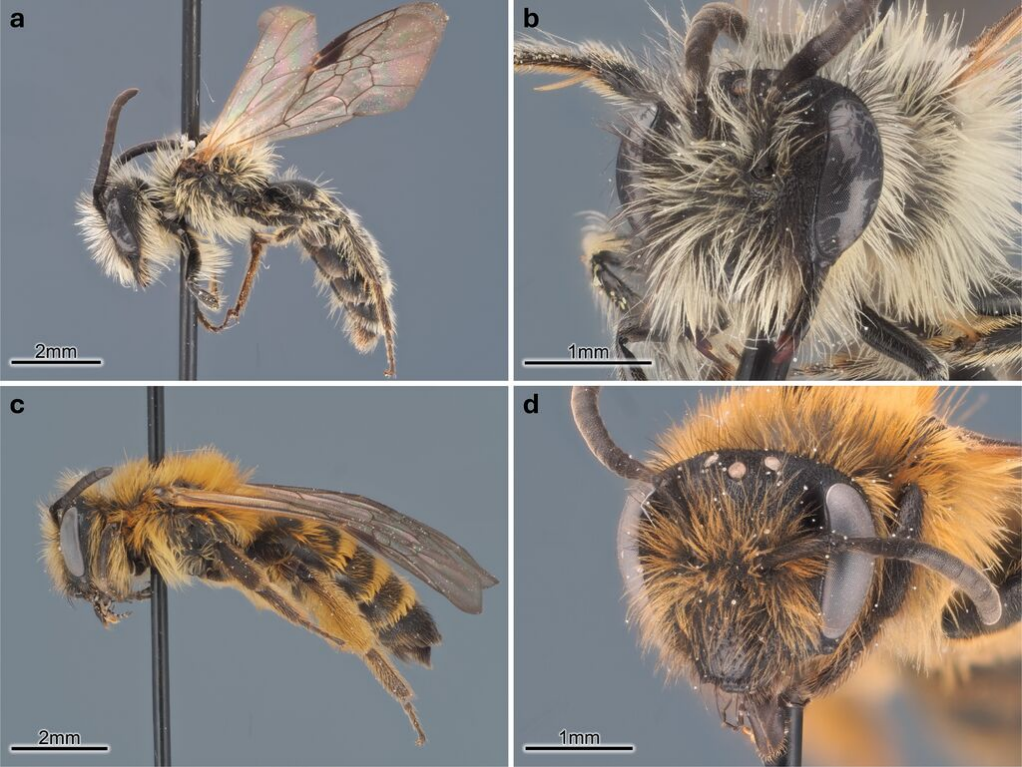
*Andrena igraeca*. **a** Habitus ♂, lateral view; **b** Head ♂, oblique view; **c** Habitus ♀, lateral view; **d** Head ♀, oblique view. Photo credit R. Santerre.

*Andrena igraeca* (Fig. 2) is a recently described species whose presence in the Hatay Province was presumed due to a record in north-west Syria (Pisanty et al. 2022). It is now confirmed to occur in Türkiye by the 13 specimens collected in three locations in Nur Mountains, near to the Hatay Province (Fig. 1d).

#### Anthophora dalmatica

Pérez, 1902

0FF602F8-6FB8-5BF8-8F90-94EAD1494E84

##### Materials

- **Type status:** Other material. **Occurrence:** recordedBy: G. Ghisbain, D. Michez & R. Santerre; sex: 2 males; occurrenceID: 9CCFBD45-A5B5-5C9F-962D-6250EB632A6D; **Location:** country: Türkiye; stateProvince: Mersin; locality: Anamur; verbatimElevation: 465 m; decimalLatitude: 36.07575; decimalLongitude: 32.63737; **Event:** eventDate: 24/04/2025

##### Distribution

South-eastern Europe (Rasmont et al. 2025), Western Asia (Boustani et al. 2021), ? Central Asia (Ascher and Pickering 2025).

##### Notes

**Figure 3.**
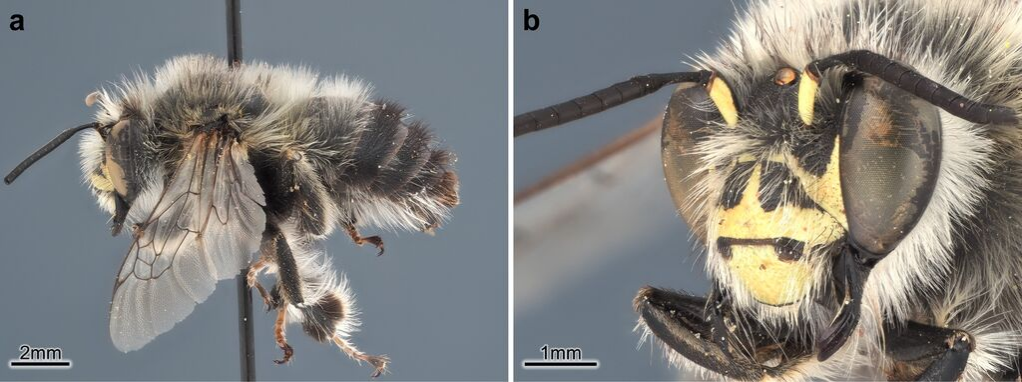
*Anthophora dalmatica* ♂. **a** Habitus, lateral view; **b** Head, oblique view. Photo credit R. Santerre.

**Figure 4.**
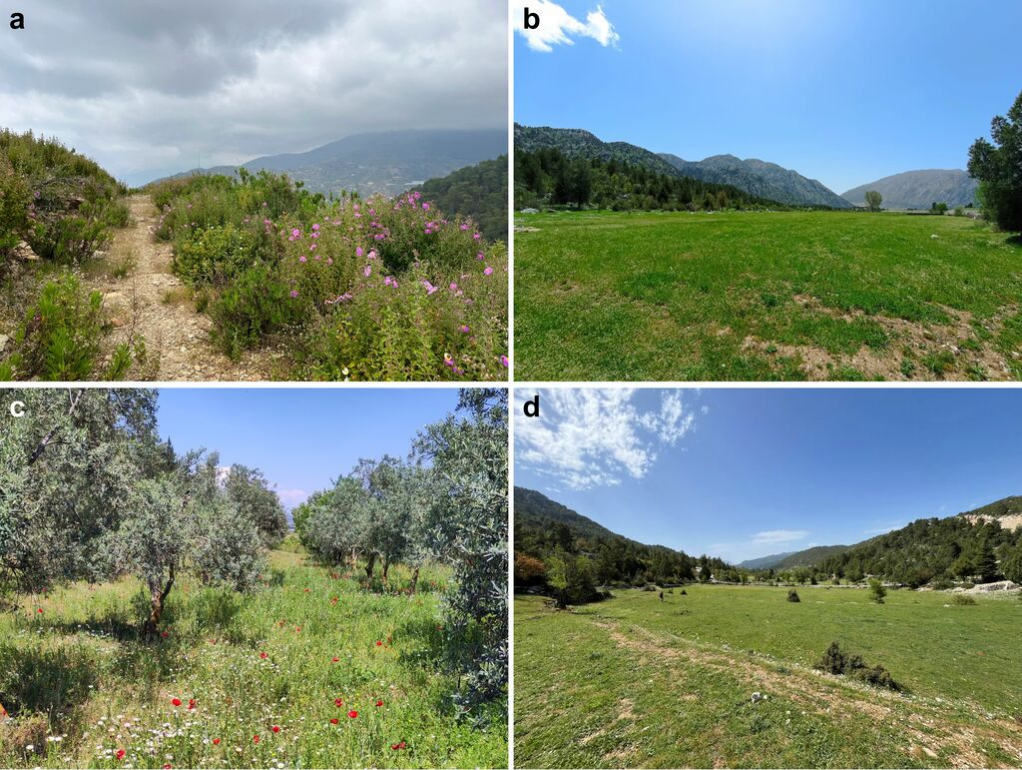
Sampling sites. **a** Shrubland dominated by *Cistus* sp. near Anamur (Mersin Province); **b** Meadow near İbradi (Antalya Province); **c** Orchard near Adana (Adana Province); **d** Meadow near Alaçeşme (Antalya Province). Photo credit G. Ghisbain (a, d), R. Santerre (b) & D. Michez (c).

Although the boundaries of the distribution of *Anthophora dalmatica* (Fig. 3) are unclear due to the unresolved taxonomy of the species-group to which it belongs, its presence in Türkiye was likely as it occurs in the surrounding areas (Boustani et al. 2021, Rasmont et al. 2025). Existing records of *A. atroalba* Lepeletier from Türkiye (e.g. Grace (2010), Ascher and Pickering (2025)) might actually pertain to this species. The two males were collected in a *Cistus*-rich shrubland near Anamur (Fig. 4a).

#### Anthophora subterranea

(Germar, 1826)

CF613A38-5209-5D9E-806D-44047E4AF809

##### Materials

- **Type status:** Other material. **Occurrence:** recordedBy: G. Ghisbain, D. Michez & R. Santerre; sex: 1 female; occurrenceID: 221BEA6A-9FE7-5A50-A84E-E2FCA2D1A61A; **Location:** country: Türkiye; stateProvince: Osmaniye; locality: Dereobası; verbatimElevation: 1926 m; decimalLatitude: 36.96265; decimalLongitude: 36.36852; **Event:** eventDate: 28/04/2025

##### Distribution

Circum-Mediterranean (Rasmont 2014, Ascher and Pickering 2025).

##### Notes

**Figure 5.**
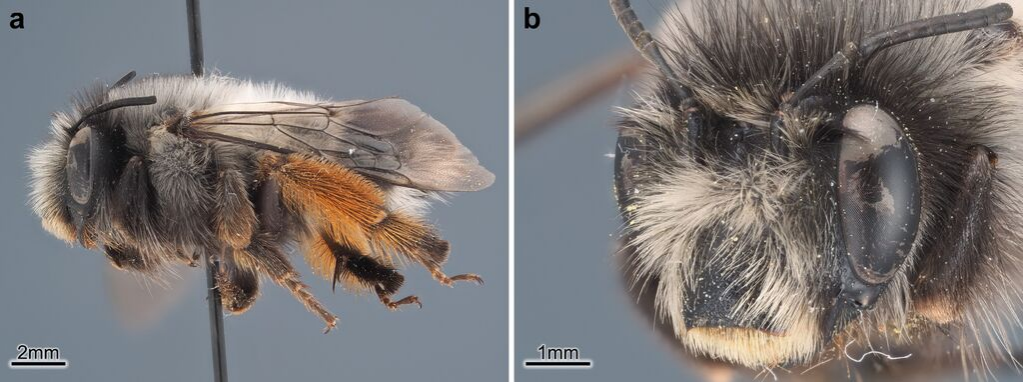
*Anthophora subterranea* ♀. **a** Habitus, lateral view; **b** Head, oblique view. Photo credit R. Santerre.

*Anthophora subterranea* (Fig. 5) is a polytypic species that has numerous synonymes (e.g. *canescens* Brullé, *lanata* Klug, *laticincta* Dours, *nigrocincta* Lepeletier, *procera* Costa), but despite being relatively common, none of these names has been reported yet from Türkiye to the best of our knowledge. The single female was collected in Nur Mountains, at the border with Hatay Province (Fig. 1d).

#### Eucera parnassia

Pérez, 1902

76B8B08D-C2D5-54A1-B6D1-7A47A6E213CE

##### Materials

- **Type status:** Other material. **Occurrence:** recordedBy: G. Ghisbain, D. Michez & R. Santerre; sex: 5 females; occurrenceID: 74231601-7183-5B50-AA70-5D689D99F435; **Location:** country: Türkiye; stateProvince: Antalya; locality: İbradi; verbatimElevation: 956 m; decimalLatitude: 37.13349; decimalLongitude: 31.47374; **Event:** eventDate: 23/04/2025

##### Distribution

South-eastern Europe (Risch et al. 2025a), Western Asia (Khodaparast and Monfared 2013, Boustani et al. 2021).

##### Notes

**Figure 6.**
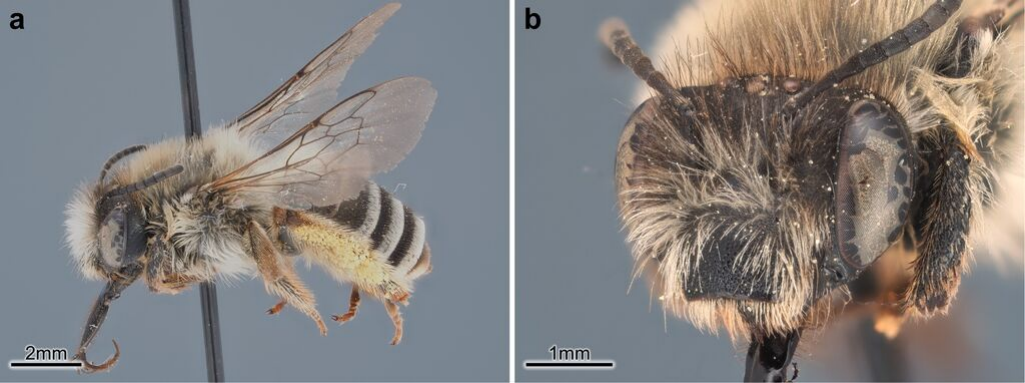
*Eucera parnassia* ♀. **a** Habitus, lateral view; **b** Head, oblique view. Photo credit R. Santerre.

*Eucera parnassia* (Fig. 6), already known from several surrounding countries (including from neighbouring Aegean islands), was likely to occur in Türkiye as well. The five females were collected in a Brassicaceae-rich meadow in a valley near İbradi (Fig. 4b).

#### Eucera parvicornis

Mocsáry, 1878

875CE333-02C7-5274-974A-CC52881AD129

##### Materials

- **Type status:** Other material. **Occurrence:** recordedBy: G. Ghisbain, D. Michez & R. Santerre; sex: 1 female, 4 males; occurrenceID: 05C290D4-8BD1-5D34-AEF2-EA02AB205CA7; **Location:** country: Türkiye; stateProvince: Mersin; locality: Geçimli; verbatimElevation: 1235 m; decimalLatitude: 36.80418; decimalLongitude: 33.32723; **Event:** eventDate: 30/04/2025

##### Distribution

Eastern and south-eastern Europe (Risch et al. 2025b), Western Asia (Ascher and Pickering 2025).

##### Notes

**Figure 7.**
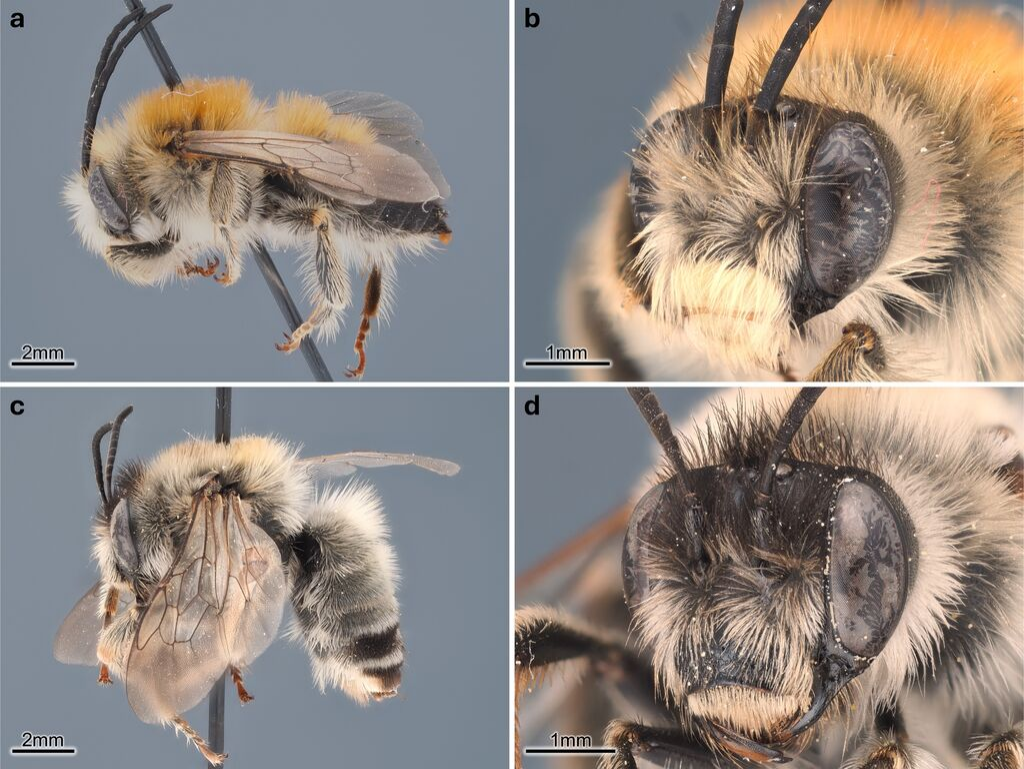
*Eucera parvicornis*. **a** Habitus ♂, lateral view; **b** Head ♂, oblique view; **c** Habitus ♀, lateral view; **d** Head ♀, oblique view. Photo credit R. Santerre.

*Eucera parvicornis* (Fig. 7) belongs to the small subgenus E. (Cubitalia), composed of eight species of which most are primarily distributed in Western Asia (Risch 1999, Aubert et al. 2024a). It is oligolectic on Boraginaceae with preference for *Anchusa* and is considered "Near Threatened" in Europe (Risch et al. 2025b, Michez et al. 2026). This species, already known from surrounding countires (including from neighbouring Aegean islands) was likely to occur in Türkiye as well.

#### Osmia jason

Benoist, 1929

573D2A35-7077-55F6-97AF-507D550B391C

##### Materials

- **Type status:** Other material. **Occurrence:** recordedBy: G. Ghisbain, D. Michez & R. Santerre; sex: 4 females; occurrenceID: 9848EAE0-9035-56AF-A0D5-1DB80EDC704E; **Location:** country: Türkiye; stateProvince: Adana; locality: Örcün; verbatimElevation: 107 m; decimalLatitude: 37.12593; decimalLongitude: 35.28524; **Event:** eventDate: 27/04/2025
- **Type status:** Other material. **Occurrence:** recordedBy: G. Ghisbain, D. Michez & R. Santerre; sex: 1 female; occurrenceID: 38A7485A-D8FB-5DEB-8C92-3FF23893B9B5; **Location:** country: Türkiye; stateProvince: Adana; locality: Sarıçam; verbatimElevation: 140 m; decimalLatitude: 37.07968; decimalLongitude: 35.36758; **Event:** eventDate: 27/04/2025

##### Distribution

South-eastern Europe, Western Asia (Müller 2022).

##### Notes

**Figure 8.**
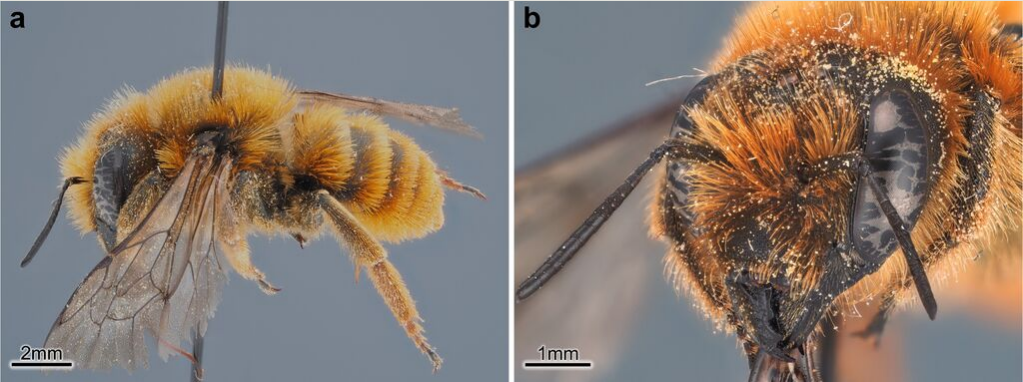
*Osmia jason* ♀. **a** Habitus, lateral view; **b** Head, oblique view. Photo credit R. Santerre.

*Osmia jason* (Fig. 8) belongs to the subgenus O. (Neosmia), whose members build their nests in empty snail shells. Its complex nesting behaviour is explained in detail by Grozdanić (1971) and Müller (2022): the cell partition is made with leaf pulp and the entry is sealed with a 3-layer final plug (leaf pulp - mineral materials - leaf pulp); the shell is plastered with leaf pulp, burried in the ground and covered with dried plant material. The five females were collected on flower-rich grasslands in orchards from two locations near Adana (e.g. Fig. 4c).

### Species threatened in neighbouring regions

#### Ammobatoides abdominalis

(Eversmann, 1852)

151C32B3-5BE4-5D82-A7C8-14209064B7AA

##### Materials

- **Type status:** Other material. **Occurrence:** recordedBy: G. Ghisbain, D. Michez & R. Santerre; sex: 1 male; occurrenceID: DC437936-45C3-5738-AB2D-DA8135AAABA7; **Location:** country: Türkiye; stateProvince: Antalya; locality: Güzelyalı; verbatimElevation: 148 m; decimalLatitude: 36.89485; decimalLongitude: 31.53827; **Event:** eventDate: 2/05/2025

##### Distribution

From Central Europe to western China and southern Siberia (Wood and Patiny 2025).

##### Notes

**Figure 9.**
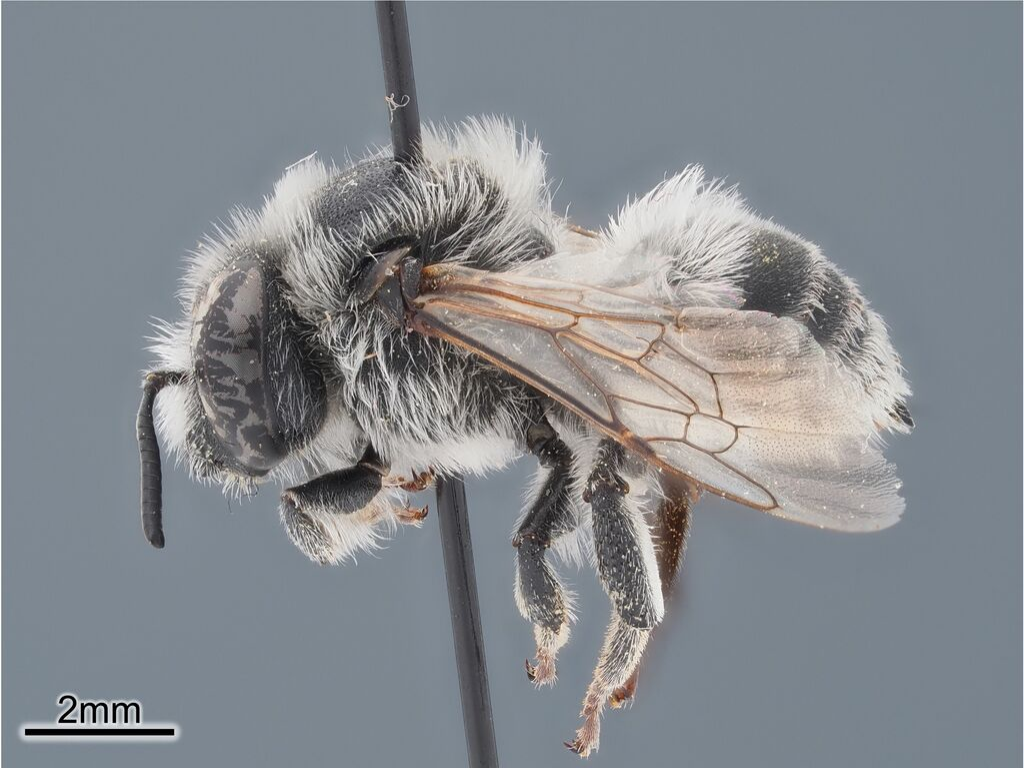
*Ammobatoides abdominalis* ♂ habitus, lateral view. Photo credit R. Santerre.

*Ammobatoides abdominalis* (Fig. 9) is considered Vulnerable in Europe (Michez et al. 2026). Three specimens of *Melitturga syriaca* Friese, a presumed host (Wood and Patiny 2025) were found at the same localion.

#### Lasioglossum laeve

(Kirby, 1802)

2D21C927-1208-5266-AE3C-F9425D4693AD

##### Materials

- **Type status:** Other material. **Occurrence:** recordedBy: G. Ghisbain, D. Michez & R. Santerre; sex: 1 female; occurrenceID: FF77BFCF-1902-5E21-B6CE-C336AF16122C; **Location:** country: Türkiye; stateProvince: Osmaniye; locality: Dereobası; verbatimElevation: 1926 m; decimalLatitude: 36.96265; decimalLongitude: 36.36852; **Event:** eventDate: 28/04/2025

##### Distribution

Europe, Western Asia and south-western Siberia (Ebmer 1995, Flaminio et al. 2025).

##### Notes

**Figure 10.**
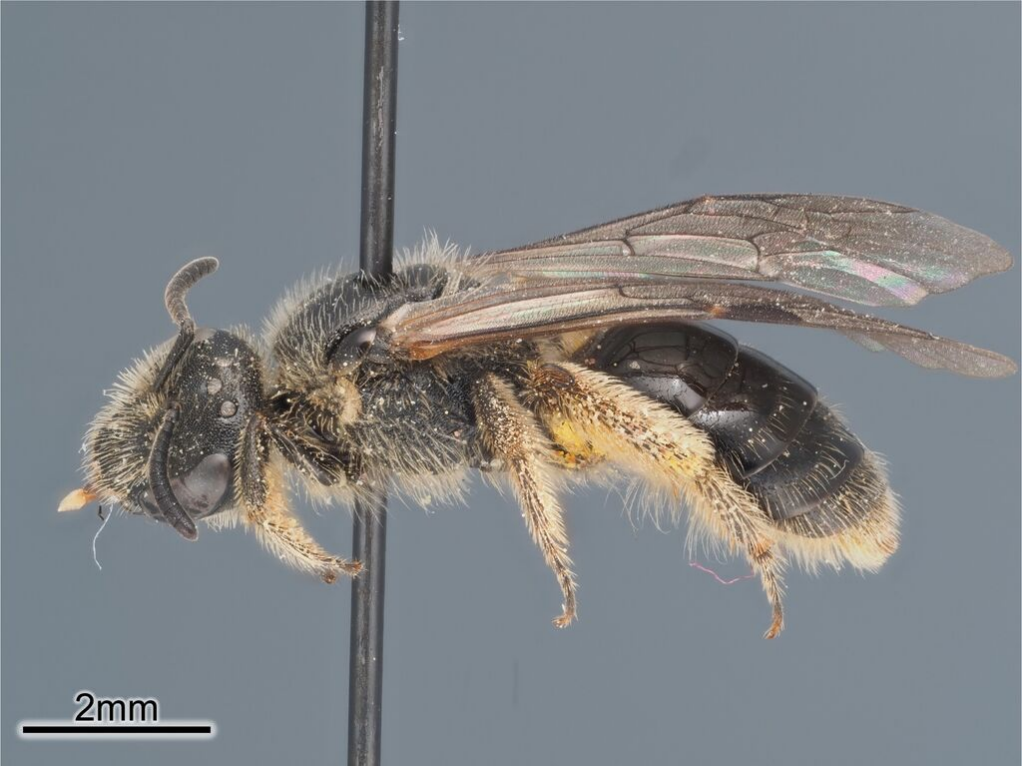
*Lasioglossum laeve* ♀ habitus, lateral view. Photo credit R. Santerre.

*Lasioglossum laeve* (Fig. 10) is considered Vulnerable in Europe (Michez et al. 2026).

### Other remarkable taxa

#### Andrena etesiaca

Warncke, 1975

F7EE578B-DA51-53FE-B091-7CD921F7D726

##### Materials

- **Type status:** Other material. **Occurrence:** recordedBy: G. Ghisbain, D. Michez & R. Santerre; sex: 1 female, 3 males; occurrenceID: C58B6830-DB74-521D-BB3E-A050A59080B3; **Location:** country: Türkiye; stateProvince: Konya; locality: Ereğli; verbatimElevation: 1112 m; decimalLatitude: 37.61552; decimalLongitude: 34.24594; **Event:** eventDate: 29/04/2025

##### Distribution

Türkiye (Pisanty and Wood 2024).

##### Notes

**Figure 11.**
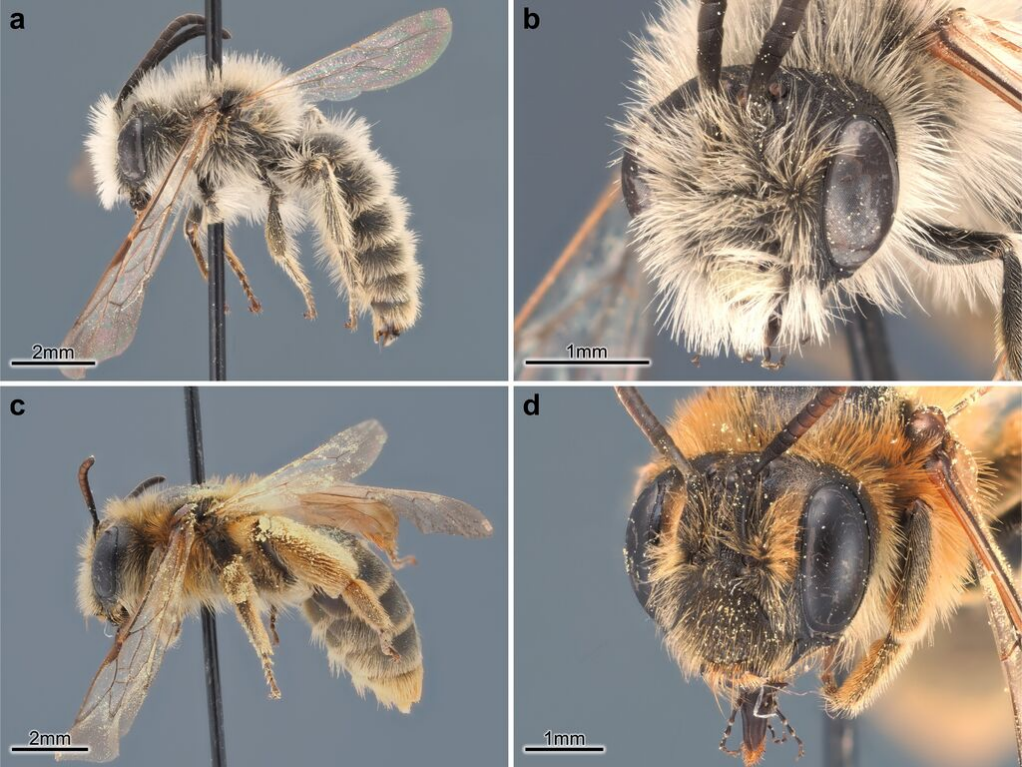
*Andrena etesiaca*. **a** Habitus ♂, lateral view; **b** Head ♂, oblique view; **c** Habitus ♀, lateral view; **d** Head ♀, oblique view. Photo credit R. Santerre.

*Andrena etesiaca* (Fig. 11) is closely related to *A. bassana* Warncke, 1969 of which it was formerly considered as a subspecies, the two being the only representatives of the subgenus A. (Vellandrena) (Pisanty and Wood 2024). *A. etesiaca* seems to be restricted to central Türkiye, where it is known from a very limited number of specimens (Hazir et al. 2014, Pisanty and Wood 2024).

#### Ceratina denesi

Terzo, 1998

3557C9F4-5E23-56C1-A4B1-7A6CF0829FA8

##### Materials

- **Type status:** Other material. **Occurrence:** recordedBy: G. Ghisbain, D. Michez & R. Santerre; sex: 4 males; occurrenceID: A0D3829F-3C36-502D-9EEE-2987F2F83FCC; **Location:** country: Türkiye; stateProvince: Antalya; locality: Alaçeşme; verbatimElevation: 782 m; decimalLatitude: 36.97435; decimalLongitude: 31.73537; **Event:** eventDate: 23/04/2025

##### Distribution

Türkiye (Özbek and Terzo 2016).

##### Notes

**Figure 12.**
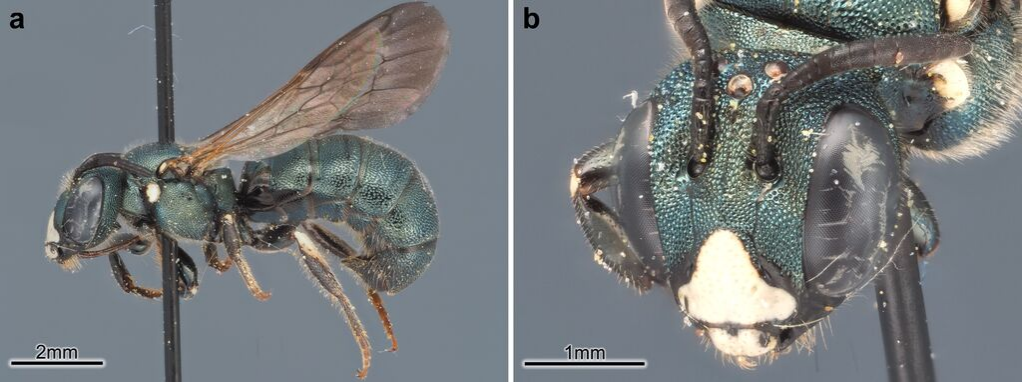
*Ceratina denesi* ♂. **a** Habitus, lateral view; **b** Head, oblique view. Photo credit R. Santerre.

*Ceratina denesi* (Fig. 12) was described, based on a single male specimen collected in Silifke (Mersin Province) in 1995 (Terzo 1998). Later, one specimen collected near Antalya in 2002 was putatively identified as the still undescribed female (Özbek and Terzo 2016). No other records of this species have been published since then. The size of the four specimens collected near Alaçeşme (Fig. 4d) is similar to *C. loewi* Gerstaecker and *C. cypriaca* Mavromoustakis, therefore larger than the holotype. However, the shape of tergum 7, the absence of concavity on apical half of the clypeus and overall blue colouration separate it from the two latter species (Terzo 1998).

#### Ceratina rasmonti

Terzo, 1998

6EB27068-C0D6-54A4-A394-7E4A7C12FA2B

##### Materials

- **Type status:** Other material. **Occurrence:** recordedBy: G. Ghisbain, D. Michez & R. Santerre; sex: 1 female; occurrenceID: 59AB6CE2-7933-55A1-8985-3411835F198B; **Location:** country: Türkiye; stateProvince: Antalya; locality: Beydiğin; verbatimElevation: 478 m; decimalLatitude: 37.01694; decimalLongitude: 31.39717; **Event:** eventDate: 23/04/2025
- **Type status:** Other material. **Occurrence:** recordedBy: G. Ghisbain, D. Michez & R. Santerre; sex: 1 female; occurrenceID: B47FDD63-18D5-517A-8B74-CFB90BC9C8DC; **Location:** country: Türkiye; stateProvince: Antalya; locality: İbradi; verbatimElevation: 956 m; decimalLatitude: 37.13349; decimalLongitude: 31.47374; **Event:** eventDate: 23/04/2025

##### Distribution

Türkiye (Özbek and Terzo 2016).

##### Notes

**Figure 13.**
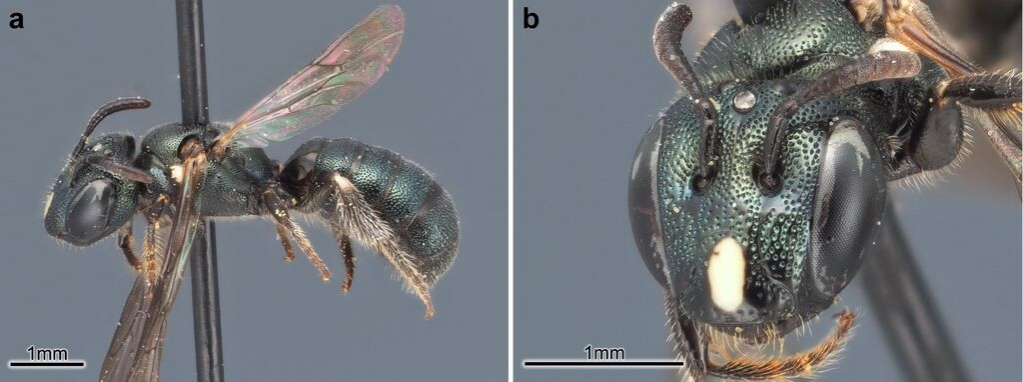
*Ceratina rasmonti* ♀. **a** Habitus, lateral view; **b** Head, oblique view. Photo credit R. Santerre.

*Ceratina rasmonti* (Fig. 13) was only known from the four females constituting the type series, collected in Erçek and Ağrı in eastern Türkiye, at an altitude of around 1,800 m (Terzo 1998, Özbek and Terzo 2016). The finding of this species in south-western Türkiye, at 478 m near Beydiğin (Fig. 1a) and 956 m near İbradi (Fig. 4b), was therefore unexpected.

**Figure 14.**
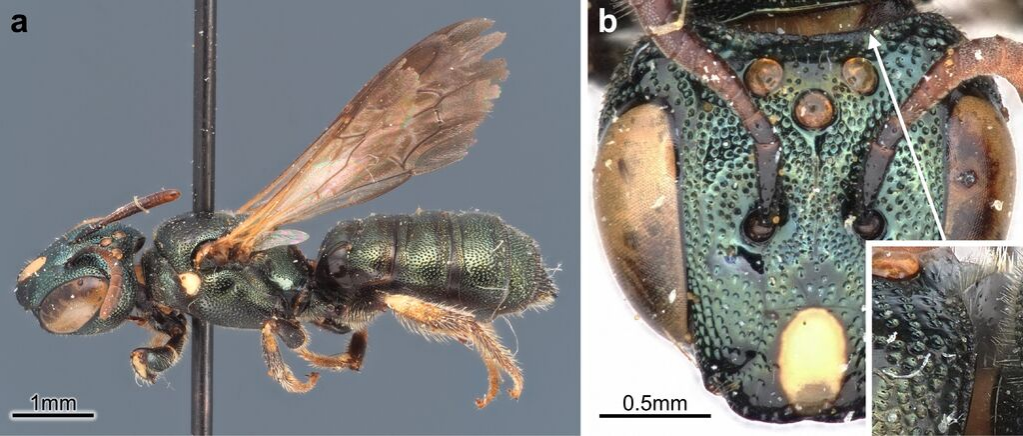
Paratype of *Ceratina rasmonti* ♀. **a** Habitus, lateral view; **b** Head in front view, with close-up on the occipital margin in lateral view. Photo credit R. Santerre.

The new specimens have the tibiae less extensively white-marked than the type specimens collected in eastern Türkiye (Fig. 14) and have been first misidentified as *C. dallatorreana* Friese, a related and much more common species.

**Figure 15.**
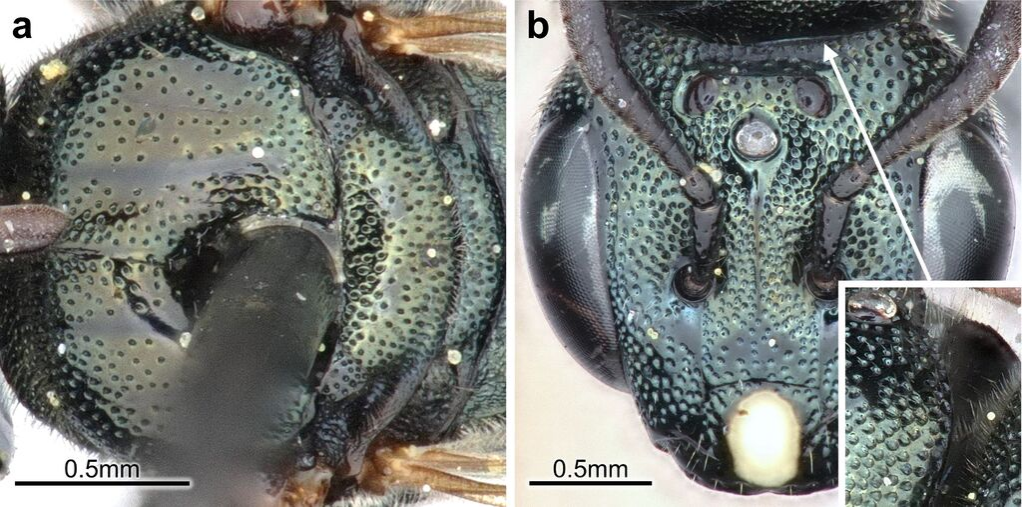
*Ceratina rasmonti* ♀. **a** Scutum; **b** Head in front view, with close-up on the occipital margin in lateral view. Photo credit R. Santerre.

**Figure 16.**
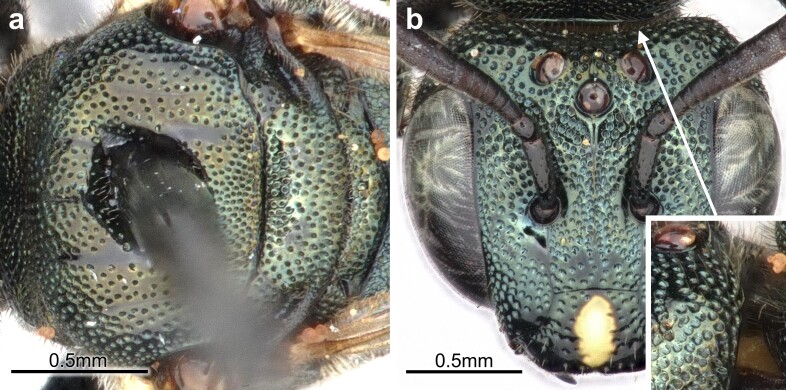
*Ceratina dallatorreana* ♀. **a** Scutum; **b** Head in front view, with close-up on the occipital margin in lateral view. Photo credit R. Santerre.

The two species could be distinguished only after a second careful examination, *C. rasmonti* having sparser punctation on scutum and between oecelli, depressed ocello-occipital area, carinate occipital margin (Figs 14, 15) and less developed wax glands on sternum 3 than *C. dallatorreana* (Fig. 16) (Terzo 1998).

This suggests that this presumably rare species might be under-recorded due to the difficulty in detecting it amongst series of *C. dallatorreana*.

## Supplementary Material

XML Treatment for Andrena igraeca

XML Treatment for Anthophora dalmatica

XML Treatment for Anthophora subterranea

XML Treatment for Eucera parnassia

XML Treatment for Eucera parvicornis

XML Treatment for Osmia jason

XML Treatment for Ammobatoides abdominalis

XML Treatment for Lasioglossum laeve

XML Treatment for Andrena etesiaca

XML Treatment for Ceratina denesi

XML Treatment for Ceratina rasmonti

1AC77915-D931-53AF-901F-ED2BCF319EE8 10.3897/BDJ.14.e191847.suppl1 Supplementary material 1 Wild bees collected in southern Türkiye in spring 2025 Data type occurrences Brief description The dataset provides spatio-temporal information for 2,070 bee specimens (Anthophila) collected in southern Türkiye, during a field expedition carried out from 23-04-2025 to 02-05-2025. File: oo_1546841.txt https://binary.pensoft.net/file/1546841 Rémi Santerre, Jordan Benrezkallah

## Figures and Tables

**Table 1.** Summary list of the taxa and number of specimens collected. The nomenclature used follows that of Ghisbain et al. (2025) for the taxa that are shared with Europe and the most recent expert-based knowledge for non-European species (marked by *).

**Family**	**Genus**	**Species**	**Number of specimens**
Andrenidae	* Andrena *	*abbreviata* Dours, 1873	14
		*afzeliella* (Kirby, 1802)	1
		cf. *alfkenella* Perkins, 1914	8
		*alfkenelloides* Warncke, 1965	3
		*bisulcata* Morawitz, 1877	2
		*brumanensis* Friese, 1899	13
		*canohirta* (Friese, 1923)	1
		*cinnamonea* Warncke, 1975 *	1
		*combinata* (Christ, 1791)	6
		*cypria* Pittioni, 1950	1
		*etesiaca* Warncke, 1975 *	4
		*exquisita* Warncke, 1975	11
		*ferox* Smith, 1847	2
		*flavipes* Panzer, 1799	122
		*floricola* Eversmann, 1852	11
		*fulvitarsis* Brullé, 1832	2
		*fuscosa* Erichson, 1835	2
		*gallinula* Warncke, 1975 *	50
		*glandaria* Warncke, 1975	39
		*helvola* (Linnaeus, 1758)	1
		*humilis* Imhoff, 1832	5
		*igraeca* Pisanty & Wood, 2022 *	13
		*impunctata* Pérez, 1895	1
		*kurda* Warncke, 1975 *	14
		*labialis* (Kirby, 1802)	1
		*labiata* Fabricius, 1781	5
		*lamiana* Warncke, 1965	3
		*lepida* Schenck, 1861	6
		*limata* Smith, 1853	1
		*magunta* Warncke, 1965	1
		*merula* Warncke, 1969	3
		*minutula* (Kirby, 1802)	3
		*monacha* Warncke, 1965	12
		*morio* Brullé, 1832	1
		*neocypriaca* Mavromoustakis, 1956	10
		*nigroaenea* (Kirby, 1802)	6
		*optata* Warncke, 1975	7
		*paradoxa* Friese, 1921 *	10
		*pareklisiae* Mavromoustakis, 1957	3
		*pilipes* Fabricius, 1781	9
		*pulicaria* Warncke, 1975 *	21
		*pyropygia* Kriechbaumer, 1873	3
		*rotundilabris* Morawitz, 1878	10
		*rugothorace* Warncke, 1965	23
		*rugulosa* Stöckhert, 1935	1
		*rusticola* Warncke, 1975 *	5
		*schlettereri* Friese, 1896	9
		*schmiedeknechti* Magretti, 1883	1
		*semirubra* Morawitz, 1876	1
		*sillata* Warncke, 1975	1
		*spreta* Pérez, 1895	90
		*taedium* Wood, 2023	7
		aff. *taraxaci* Giraud, 1861	31
		*toelgiana* Friese, 1921 *	2
		*tringa* Warncke, 1973	2
		*truncatilabris* Morawitz, 1877	2
		*tscheki* Morawitz, 1872	1
		>aff. *unicincta* Friese, 1899 *	2
		*ventricosa* Dours, 1873	1
		*vetula* Lepeletier, 1841	1
		*viridescens* Viereck, 1916	6
		*volka* Warncke, 1969 *	1
		*wilhelmi* Schuberth, 1995	5
		spp.	26
	* Camptopoeum *	*variegatum* (Morawitz, 1876)	4
	* Melitturga *	*syriaca* Friese, 1899	13
	* Panurginus *	*corpanus* (Warncke, 1972)	9
	* Panurgus *	*oblitus* Warncke, 1972	10
Apidae	* Amegilla *	*albigena* (Lepeletier, 1841)	2
		*quadrifasciata* (de Villers, 1789)	5
		spp.	7
	* Ammobatoides *	*abdominalis* (Eversmann, 1852)	1
	* Anthophora *	*agama* Radoszkowski, 1869	14
		*crinipes* Smith, 1854	54
		*dalmatica* Pérez, 1902	2
		*nigriceps* Morawitz, 1886	5
		cf. *plumipes* (Pallas, 1772)	23
		*rogenhoferi* Morawitz, 1872	13
		*rubricrus* Dours, 1869 *	1
		*subterranea* (Germar, 1826)	1
		spp.	5
	* Bombus *	*hortorum* (Linnaeus, 1761)	1
		gr. *terrestris* (Linnaeus, 1758)	43
	* Ceratina *	*bifida* Friese, 1900 *	2
		*bispinosa* Handlirsch, 1889	4
		*chrysomalla* Gerstaecker, 1869	3
		*cucurbitina* (Rossi, 1792)	2
		*dallatorreana* Friese, 1896	3
		*denesi* Terzo, 1998 *	4
		*mandibularis* Friese, 1896	20
		*moricei* Friese, 1899	3
		*nigroaenea* Gerstaecker, 1869	5
		*rasmonti* Friese, 1899 *	2
		*schwarzi* Kocourek, 1998	2
	* Eucera *	*albofasciata* Friese, 1895	12
		*caerulescens* Friese, 1899	2
		*cypria* Alfken, 1933	1
		*dafnii* Dorchin, 2019	1
		*dalmatica* Lepeletier, 1841	1
		*digitata* Friese, 1896	24
		*dimidiata* Brullé, 1832	2
		*gaullei* Vachal, 1907	28
		*kullenbergi* Tkalců, 1978	5
		*laxiscopa* Alfken, 1935	9
		*mediterranea* Friese, 1896	2
		*nigrescens* Pérez, 1879	3
		*nigrilabris* Lepeletier, 1841	26
		*parnassia* Pérez, 1902	5
		*parvicornis* Mocsáry, 1878	5
		*plumigera* (Kohl, 1905)	22
		*pseudeucnemidea* Risch, 1997	5
		*punctulata* Alfken, 1942	9
		*seminuda* Brullé, 1832	4
		*tricincta* Erichson, 1835	10
		*vulpes* Brullé, 1832	11
		spp.	21
	* Eupavlovskia *	*obscura* (Friese, 1895)	1
	* Habropoda *	*tarsata* (Spinola, 1838)	4
	* Nomada *	*basalis* Herrich-Schäffer, 1839	2
		*cherkesiana* Mavromoustakis, 1955	1
		*flavoguttata* (Kirby, 1802)	3
		*fucata* Panzer, 1798	2
		*furvoides* Stöckhert, 1943	1
		*goodeniana* (Kirby, 1802)	6
		*guttulata* Schenck, 1861	1
		*lucidula* Schwarz, 1967	1
		*mutica* Morawitz, 1872	1
		*nobilis* Herrich-Schäffer, 1839	1
		*pallispinosa* Schwarz, 1967	1
		*panzeri* Lepeletier, 1841	9
		*radoszkowskii* Łoziński, 1922	5
		*schmidti* Schwarz, Smit & Ockermüller, 2020 *	4
		>aff. *sheppardana* (Kirby, 1802)	4
		*striata* Fabricius, 1793	2
	* Xylocopa *	*iris* (Christ, 1791)	6
		*pubescens* Spinola, 1838	17
		*violacea* (Linnaeus, 1758)	10
Colletidae	* Colletes *	*cariniger* Pérez, 1903	2
		*mlokossewiczi* Radoszkowski, 1891	2
		*similis* Schenck, 1853	1
	* Hylaeus *	*brevicornis* Nylander, 1852	1
		*communis* Nylander, 1852	1
		*cornutus* Curtis, 1831	2
		*damascenus* (Magretti, 1890) *	3
		*imparilis* Förster, 1871	5
		*incongruus* Förster, 1871	26
		*intermedius* Förster, 1871	1
		*leptocephalus* (Morawitz, 1870)	1
		*lineolatus* (Schenck, 1861)	1
		*orientalicus* (Warncke, 1981)	1
		*punctatus* (Brullé, 1832)	12
		*sidensis* (Warncke, 1981)	1
		*stellatus* (Warncke, 1992)	5
		*taeniolatus* Förster, 1871	5
		*trinotatus* (Pérez, 1895)	3
		*variegatus* (Fabricius, 1798)	1
Halictidae	* Ceylalictus *	*variegatus* (Olivier, 1789)	9
	* Dufourea *	*cypria* Mavromoustakis, 1952	2
	* Halictus *	*cochlearitarsis* (Dours, 1872)	4
		*graecus* Blüthgen, 1933	2
		*quadricinctus* (Fabricius, 1777)	1
		*resurgens* Nurse, 1903	16
		*sexcinctus* (Fabricius, 1775)	4
		*tetrazonianellus* Strand, 1909	7
		spp.	29
	* Lasioglossum *	*adaliae* (Blüthgen 1923) *	1
		*aegyptiellum* (Strand, 1909)	3
		*aeratum* (Kirby, 1802)	1
		*bicallosum* (Morawitz, 1873)	2
		*bluethgeni* Ebmer, 1971	2
		*calceatum* (Scopoli, 1763)	7
		*clypeiferellum* (Strand, 1909)	1
		*cristula* (Pérez, 1896)	2
		*damascenum* (Pérez, 1910)	1
		*dolichocephalum* (Blüthgen, 1923)	5
		*fallax* (Morawitz, 1874)	1
		*interruptum* (Panzer, 1798)	4
		*laeve* (Kirby, 1802)	1
		*laevigatum* (Kirby, 1802)	5
		*laticeps* (Schenck, 1870)	3
		*lativentre* (Schenck, 1853)	1
		*leucozonium* (Schrank, 1781)	30
		*lineare* (Schenck, 1870)	14
		*littorale* (Blüthgen, 1924)	19
		*malachurum* (Kirby, 1802)	26
		*marginatum* (Brullé, 1832)	223
		*montifringillum* Warncke 1984 *	2
		*obscuratum* (Morawitz, 1876)	2
		*pallens* (Brullé, 1832)	1
		*parvulum* (Schenck, 1853)	1
		*pauperatum* (Brullé, 1832)	7
		*pauxillum* (Schenck, 1853)	1
		*pseudocaspicum* (Blüthgen, 1923)	1
		*pygmaeum* (Schenck, 1853)	2
		*rupestre* (Warncke 1984) *	1
		*tadschicum* (Blüthgen 1929) *	4
		*transitorium* (Schenck, 1870)	6
		*tricinctum* Ebmer 1972	8
		*villosulum* (Kirby, 1802)	2
		*xanthopus* (Kirby, 1802)	17
		spp.	12
	* Nomiapis *	*diversipes* (Latreille, 1806)	4
		*monstrosa* (Costa, 1861)	2
	* Rophites *	*nigripes* Friese 1902 *	3
	* Seladonia *	*cephalica* (Morawitz, 1874)	3
		*subaurata* (Rossi, 1792)	1
		sp.	1
	* Sphecodes *	*barbatus* Blüthgen, 1923	2
		*monilicornis* (Kirby, 1802)	2
		cf. *nomioidis* Pesenko, 1979	4
		*zangherii* Noskiewicz, 1931	1
	* Thrincohalictus *	*prognatus* (Pérez, 1912)	2
Megachilidae	* Anthidiellum *	*strigatum* (Panzer, 1805)	4
	* Anthidium *	*cingulatum* Latreille, 1809	1
	* Chelostoma *	*emarginatum* (Nylander, 1856)	2
		*longifacies* Müller, 2012	5
		*rapunculi* (Lepeletier, 1841)	1
		spp.	11
	* Coelioxys *	*afer* Lepeletier, 1841	1
		*haemorrhoa* Förster, 1853	1
	* Heriades *	*rubicola* Pérez, 1890	28
	* Hoplitis *	*acuticornis* (Dufour & Perris, 1840)	2
		*annulata* (Latreille, 1811)	5
		*antalyae* Tkalců, 2000	6
		>cf. *corcyraea* (Tkalců, 1979)	8
		*limassolica* (Mavromoustakis, 1937)	9
		*lysholmi* (Friese, 1899)	1
		*pallicornis* (Friese, 1895)	12
		*yermasoyiae* (Mavromoustakis, 1938)	11
	* Megachile *	*lefebvrei* (Lepeletier, 1841)	1
		*manicata* Giraud, 1861	1
		*montenegrensis* Dours, 1873	4
		*parietina* (Geoffroy, 1785)	2
	* Osmia *	*andrenoides* Spinola, 1808	1
		*bicornis* (Linnaeus, 1758)	5
		*brevicornis* (Fabricius, 1798)	1
		*caerulescens* (Linnaeus, 1758)	7
		*cephalotes* Morawitz, 1870	1
		*erythrogastra* Ferton, 1905	2
		*hellados* van der Zanden, 1984	5
		*jason* Benoist, 1929	5
		*ligurica* Morawitz, 1868	8
		*mustelina* Gerstaecker, 1869	1
		*nana* Morawitz, 1873	4
		*rhodoensis* (van der Zanden, 1983)	3
		*rufohirta* Latreille, 1811	3
		*scutellaris* Morawitz, 1868	12
		*spinigera* Latreille, 1811	1
		*subcornuta* Morawitz, 1875	1
		*submicans* Morawitz, 1870	1
		*sybarita* Smith, 1853	3
		*versicolor* Latreille, 1811	2
		*viridana* Morawitz, 1874	2
	* Protosmia *	*longiceps* (Friese, 1899)	4
		*tiflensis* (Morawitz, 1876)	1
	* Pseudoanthidium *	*cribratum* (Morawitz, 1875) *	1
	* Rhodanthidium *	*septemdentatum* (Latreille, 1809)	9
	* Stelis *	*minuta* Lepeletier & Audinet-Serville, 1825	1
		*nasuta* (Latreille, 1809)	1
		*signata* (Latreille, 1809)	1
	* Stenoheriades *	*coelostoma* (Benoist, 1935)	28
Melittidae	* Dasypoda *	*tubera* Warncke, 1973 *	35
Total			2070
